# PDMS samples characterization with variations of synthesis parameters for tunable optics applications

**DOI:** 10.1016/j.heliyon.2019.e03064

**Published:** 2019-12-18

**Authors:** Angel S. Cruz-Félix, Agustin Santiago-Alvarado, Josimar Márquez-García, Jorge González-García

**Affiliations:** a Physics and Mathematics Institute, Technological University of the Mixteca, Carretera a Acatlima km 2.5, Huajuapan de León, OAX, C.P. 69000, México; b Division of Postgraduate Studies, Technological University of the Mixteca, Carretera a Acatlima km 2.5, Huajuapan de León, OAX, C.P. 69000, México

**Keywords:** Optics, Materials science, PDMS characterisation, Elastomer synthesis, Multi-layer biconic surface, Mechanical properties, Physical properties

## Abstract

PDMS has become a frequently used material in the elaboration of optical components such as: variable focal length liquid lenses, optical waveguides, solid elastic lenses, etc. In this work we describe the elaboration of PDMS samples, and we present the physical and optical properties of the material when a variation on its synthesis parameters (mixture ratio of base: curing agent, curing temperature and curing time) is implemented during their elaboration. Tensile and compressive tests were carried out to obtain the corresponding stress-strain curves of the material, and UV-Vis spectroscopy was applied to obtain transmittance and absorbance curves of the samples. A variation of the refractive index of the samples was observed and homogeneity of the samples was studied with the Raman spectra obtained from the samples. Results of the characterization determined the appropriate synthesis parameters for the elaboration of a tunable refractive surface for potential applications in artificial vision.

## 1 Introduction

The field of optical instrumentation has evolved due to new technological requirements and the introduction of new free-form optics components which include optical microcomponents, tunable lenses, gradient index optical components etc. [1, 2, 3, 4, 5]; new processes for fabrication are needed since the techniques and materials currently used are limited to meet demand [6, 7, 8]. Thus, the implementation of new materials in the manufacture of optical components is crucial [9, 10], e.g. polydimethylsiloxane, better known as PDMS (Sylgard 184), is an elastomer that has become attractive in diverse technological applications, due to its high flexibility, easy handling, high transparency, low weight, non-toxicity and low cost; also, the use of PDMS reduces both, time and complexity in the elaboration of components [11, 12]. Due to its excellent properties and optomechanical features, in recent years this elastomer has been employed to develop coatings of microelectronic components, valves, detectors, filters, the manufacturing of liquid lenses with variable focal length, solid elastic lenses, and elements for microelectromechanical systems (MEMS) [13, 14, 15, 16, 17, 18].

Reports of the study of mechanical, chemical and optical properties of PDMS are found in the literature for cases where specific synthesis parameters are applied in the material, such as the mixture ratio (base: curing agent), temperature gradients and different curing times, composite materials, etc. [19, 20, 21, 22, 23, 24].

Regarding its mechanical characterization, the measurements of its tension, compression and shear moduli, Poisson ratio, and density with different mixture ratios (base: curing agent) have been previously reported for the manufacture of devices for microfluidics applications [19, 25, 26]. In this direction, studies have shown that curing the material at high temperatures (above the recommended temperature reported by the supplier) affects the mechanical properties of PDMS components as its decomposition initiates within the range of 300–310 °C [21].

In the case of optical characterization of PDMS, several studies have reported measurements of its refractive index, thickness, homogeneity, peaks of vibrational modes, absorption and transmittance spectral range and Raman spectrum of Si- molecules when varying its synthesis parameters for waveguide and microchannels applications [27, 28, 29, 30]. Also, it has been found that when mechanical tension is applied onto the material, small variations on its refractive index are induced [31].

Although reports already exist regarding measurements of optical and mechanical properties for certain mixture ratios (base: curing agent), temperatures and curing times, it is necessary to generate a compilation of a set of mechanical and optical properties with variation of its synthesis parameters (mixture ratio, temperature and curing times), to be used in potential applications in the field of adaptive optics, refractive systems and optical elements with gradient index distributions.

In this direction, in recent years we have been working in the characterization of significant properties of PDMS samples (Sylgard 184 from Dow Corning) for tunable optics applications [32] and in this work we present an extension of our study, in particular we present a physical and chemical characterization of PDMS samples and their manufacturing process is described; as stated above, a compilation of relevant mechanical and optical properties is needed to manufacture tunable refractive optical elements (elements capable of modifying their optical parameters when mechanical stimulus is applied) [33, 34].

## 2 Materials and method

### 2.1 PDMS synthesis

The synthesis of the elastomer PDMS is a straightforward process when the user follows the recommended instructions of the supplier. The manufacturing kit of PDMS consists of two components, a viscous base and a liquid curing agent (catalyst). When the components are combined, a cured mixture is generated [35].

A coded matrix was established of the synthesis parameters variation for the PDMS samples as seen in Table 1 (mixture ratio, curing time and temperature); mechanical and optical properties were measured for all elaborated samples. To accomplish this, 20 samples for compressive tests were generated, 20 for tensile tests and 20 more for optical tests. A total of 60 samples were produced for the characterization of each mixture ratio within a selected range.

**Table 1:** Synthesis parameters variations for PDMS samples (3 samples by code and curing temperature).

Code	Mixture ratio (base: catalyst)			
M1	10:1			
M2	10:1.25			
M3	10:1.5			
M4	10:1.75			
M5	10:2			
**Curing Time/Temperature [min/°C]**	30/100	18/150	15/200	10/240

Synthesis parameters shown in Table 1 were selected from the supplier's recommended values and from results previously published in literature [17, 19, 27, 36, 37, 38, 39, 40]; although, an increase in the amount of polymer base is observed in other authors' works, in this study we use an increase in the amount of catalyst. The employed curing temperatures are in the range of 100–200 °C, moreover, an additional temperature of 240 °C with a curing time of 10 min, that was reported by Liu [21], was added to our study. Coded names for referring to the samples are employed throughout this document (see Table 1).

### 2.2 Materials

The equipment and materials used for the synthesis and manufacture of PDMS samples are: a kit of polymer base and a catalyst (curing agent), a beaker (50 ml), a glass stirrer, an OHAUS precision balance model AX423/E, an electric muffle JEIO TECH model OF -12 and aluminum molds for each sample.

Three different types of molds were designed and elaborated to manufacture the PDMS samples; one mold was used to fabricate compressive tests' samples, another served for tensile tests' specimens and one more for optical tests' samples. The molds were designed in commercial software and manufactured in 6061 aluminum. It has been previously reported that the material of the mold in contact with the polymerizing PDMS has a certain influence in the refractive index distribution at the boundary, however, the changes introduced to the refractive index distribution are of the order of 10^−4^ and are not significant for our purpose [24].

**Figure 1:**
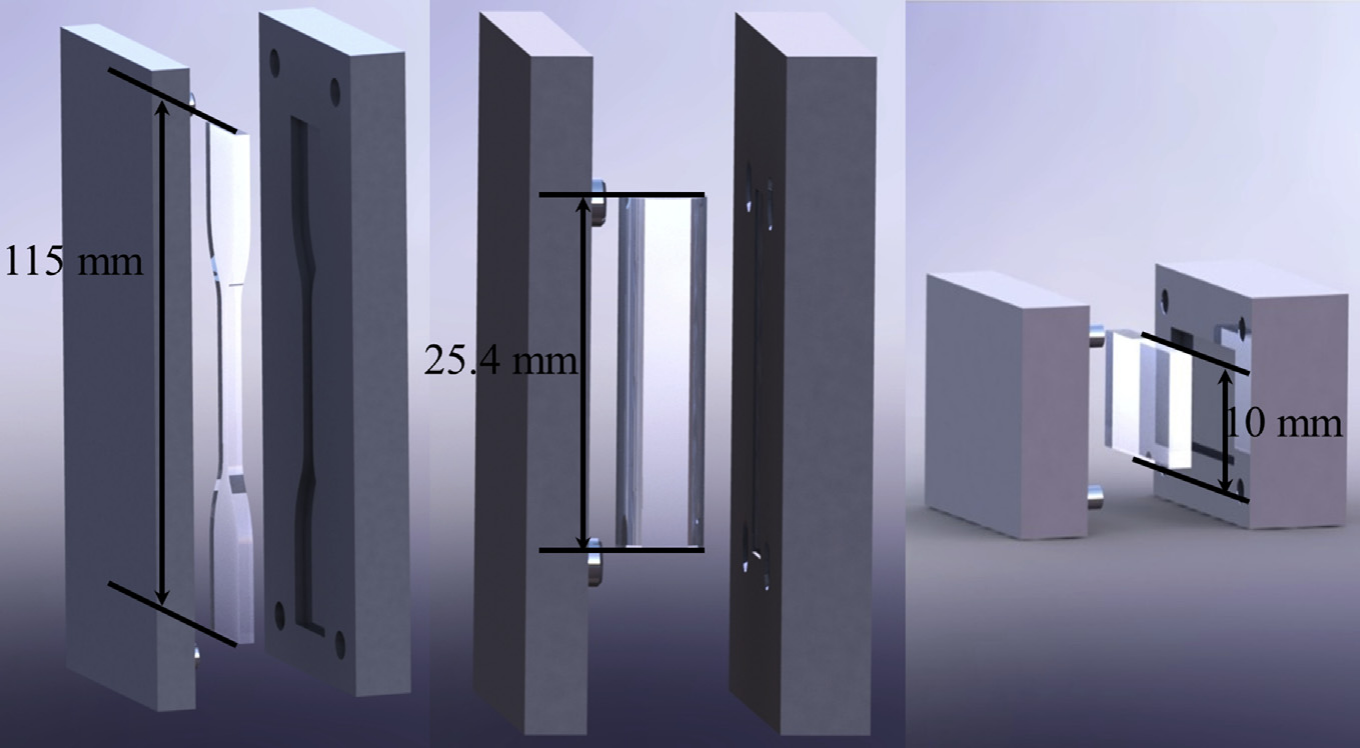
Design of the samples and molds for the elaboration of PDMS samples with variation on their synthesis parameters.

In Figure 1 we show the designed molds; on the left: mold used to manufacture tensile samples (115 mm long, 3.4 mm thick with a cylindrical shape) following the standard ASTM D412; center: mold developed to manufacture cylindrical specimens for compressive tests (25.4 mm long with a diameter of 12.7 mm) according to standard ASTM D695; right: prismatic mold used for optical tests (3mm thick, 10 mm wide and 20 mm long with rectangular shape) [41, 42].

### 2.3 Elaboration of PDMS samples

**Figure 2:**
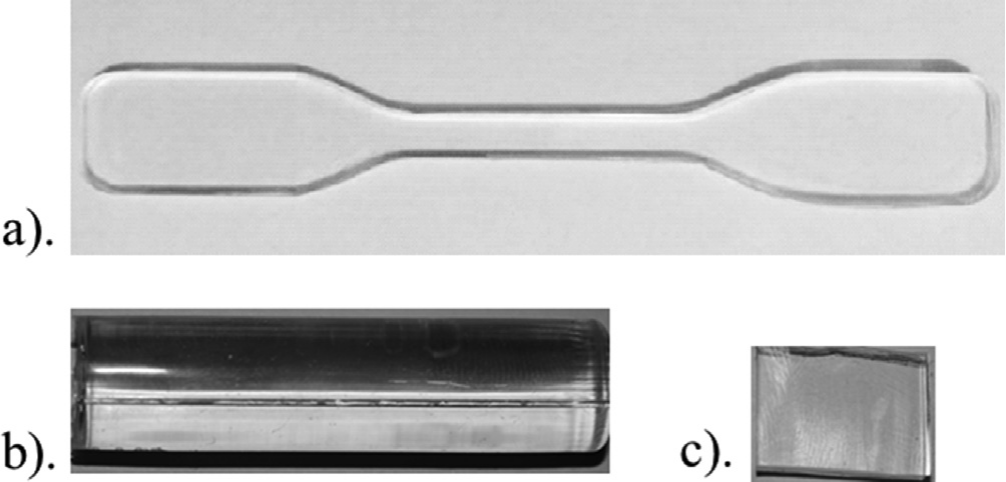
PDMS manufactured samples for: a) tensile tests, b) compressive tests and c) optical tests.

The methodology to manufacture PDMS samples consists of several steps: firstly, a beaker is tared in an analytical precision balance, i.e., its weight is previously nullified; secondly, components are manually mixed for 5 min until a homogenous phase is obtained; the weight of the components and their proportions are shown in Table 1. The obtained mixture is deposited on both faces of the mold, resting horizontally for approximately 40 min until no air bubbles are visible within the mixture; later, both parts of the mold are joined and placed in a vertical position for 2 h, to eliminate remaining air bubbles generated by the joining of the molds. Finally, in accordance with Table 1, the molds are placed inside an electric oven for curing process, the parts of the molds are separated and the PDMS specimens are obtained (see Figure 2).

### 2.4 Mechanical tests

Tensile and compressive tests were performed using a universal testing machine model Autograph AG-IS [43]. Each test was performed to obtain the mechanical properties of the samples under specific standards for repeatability accuracy.

The ASTM D412 standard was adopted for tensile tests; it is stated that bowtie-type samples must be used. The initial length was recorded and subsequently the device stretched the samples at a constant elongation speed of 200 mm/min until the rupture of samples [41]. Unitary stress-strain curves were obtained from this test, as well as its Young's modulus and the elastic limit of each sample from Table 1.

For compressive tests, the ASTM D695 standard was adopted, and cylindrical samples were placed between two parallel circular plates which compressed the samples at a constant speed of 0.08 mm/s. From this, stress-strain curves and compressive modulus were obtained, and we considered a longitudinal deformation of 25% to obtain Poisson's ratio [42].

### 2.5 Optical tests

Refractive indices of each sample were experimentally measured with an Abbe projection refractometer model WY1A from Xintian Fine Optical Instrument Corporation, that uses a lamp as illumination source with a wavelength of 589 nm and it is designed to obtain the bulk refractive index of a transparent solid. The measurement process of refractive index consisted of the following: the bottom face of the samples is covered with oil having well-known defined properties, then a reference block with a refractive index of 1.536 is employed for the device calibration, and finally the PDMS samples are placed one by one for their measurement.

Transmittance and absorbance spectra of PDMS samples were obtained using a UV-Vis spectrometer model Unicam UV 300 that employs a beam within a 200–1100 nm wavelength range [44]. The scanning mode of the device was configured to obtain the spectra, and the features of the experiment were introduced: type of experiment (transmittance or absorbance), sweep range, and the number of cycles. Then, a sweep of the base line was made to calibrate the spectrometer, and the sample was placed in the tray of the instrument.

An OCT system model Spectra Radar 930 from Thorlabs® was used. The instrument works with a wavelength of 930 ± 5 nm, spectral bandwidth of 100 ± 5 nm, optical power of 2 mW and an image depth of 1.60 mm. OCT shows the profile and homogeneity of the samples in a qualitative way, the samples were swept in horizontal direction.

Raman spectroscopy offers quantitative and/or structural chemical information from groups of chemical elements present in the material under test, the vibrational modes and the orientation of polymers chains [14, 45]. Raman spectroscopy tests were performed to the PDMS samples, a Raman spectrometer from Ocean Optics was used with a 785 nm wavelength laser as an excitation source and a two-fiber test probe (one for collection and the other for illumination of 200 and 90 μm respectively).

## 3 Results

### 3.1 Mechanical characterization

**Figure 3:**
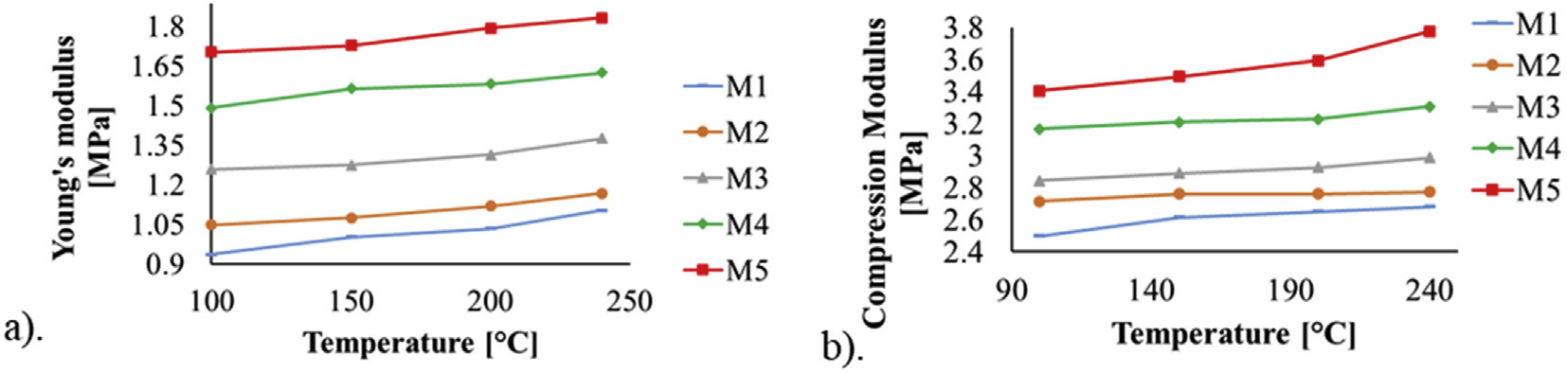
Graphs of the mechanical moduli of the samples of PDMS for: a) tension and b) compression.

Since the elasticity of the material is determined by its Young's modulus, which basically indicates the force needed to deform a piece of material, stress-strain curves were obtained from the tensile and compressive tests, and through these data the compression and tension moduli for each PDMS sample were obtained as shown in Figure 3.

With the data from the mechanical tests, the Poisson's ratio *ν*, was then calculated. To do this, a Vernier scale was used and a longitudinal guide deformation of 6.25 mm (25% length) was defined to measure the transverse deformation [46]. The transverse deformation of each sample was recorded, and an average of 1.525 mm was obtained, which is equivalent to 12% of transverse deformation. The resultant Poisson ratio is *ν* = 0.48; this value is within the range of 0.46–0.5, which has been reported in the literature for PDMS [47, 48].

**Figure 4:**
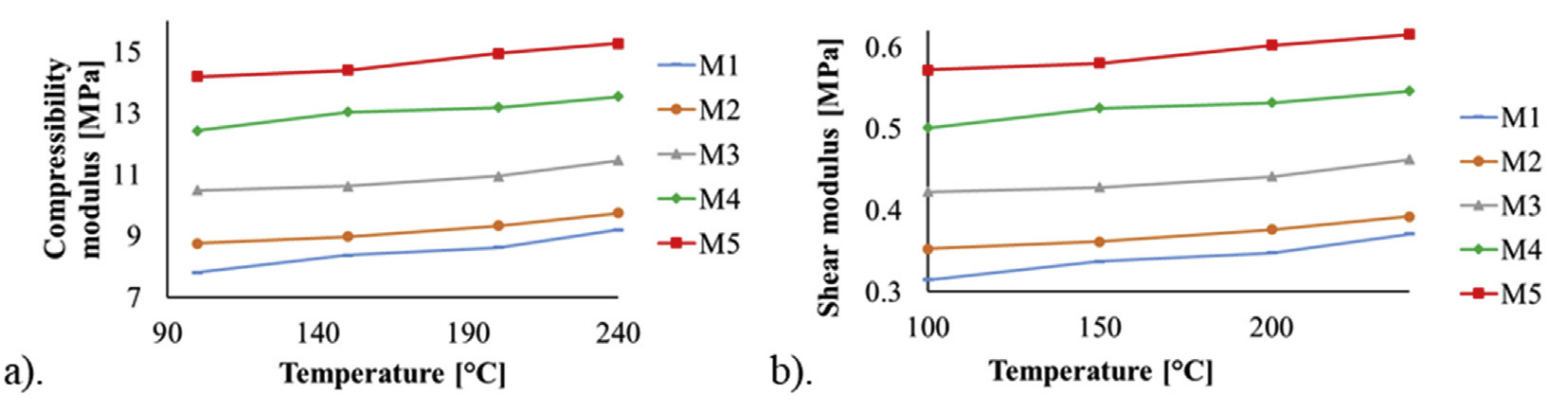
a) Compressibility modulus and, b) shear modulus of the PDMS samples.

**Figure 5:**
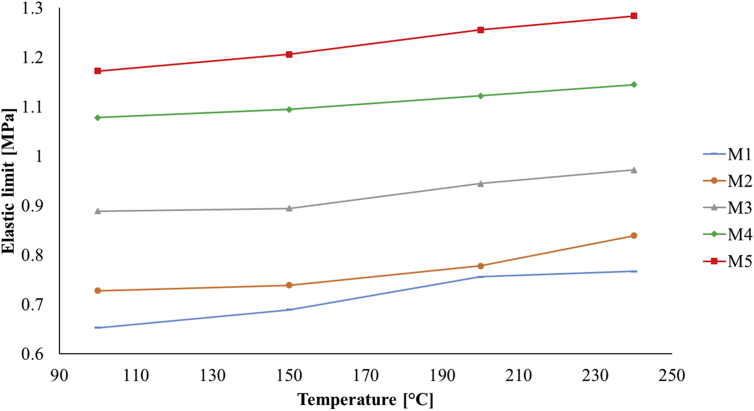
Graphs of the elastic limits of PDMS samples.

The shear modulus *G* was obtained using the definition: G=E/2(1+v), where *E* is the elastic modulus and ν is the Poisson's ratio [49]. The results are shown in Figure 4 with the compressibility modulus, where a similar behavior is observed as to the curves of tension and compression moduli. Also, from the unitary stress-strain curves, it was possible to establish the elastic limit of each PDMS sample (see Figure 5).

A genetic algorithm strategy was implemented at this point to perform a curve fitting to the results obtained for the elastic modulus, and we found that the polynomial function that describes its behavior as a function of temperature and mixture ratio of the components reads as

(1) $$E\left(T,C\right)=a_{1}T+a_{2}C+a_{3}\left(T^{2}+C^{2}\right)+a_{4}{\left(T^{2}+C^{2}\right)}^{2}+a_{5}C\left(T^{2}+C^{2}\right)+a_{6}\left(T^{2}+3C^{2}\right)+a_{7}T\left(T^{2}+C^{2}\right)+a_{8}C\left(3T^{2}+c^{2}\right)+a_{9},$$

where *E* is the elastic modulus in Pa, *T* is the temperature in °C and *C* is the components mixture ratio with dimensionless units as defined in Table 1; coefficients are: *a*_*1*_ = 0.892164618 Pa/°C, *a*_*2*_ = 4.09731434×10^−5^ Pa, *a*_*3*_ = -5.03166184×10^−5^ Pa/°C^2^, *a*_*4*_ = -3.27099606×10^−11^ Pa/°C^4^, *a*_*5*_ = 1.65817258×10^−8^ Pa/°C^2^, *a*_*6*_ = 1.56246337×10^−9^ Pa/°C^2^, *a*_*7*_ = -8.95471208×10^−8^ Pa/°C^3^, *a*_*8*_ = 5.06320175×10^−5^ Pa/°C^2^, and *a*_*9*_ = 2.58412164 Pa.

### 3.2 Optical characterization

**Figure 6:**
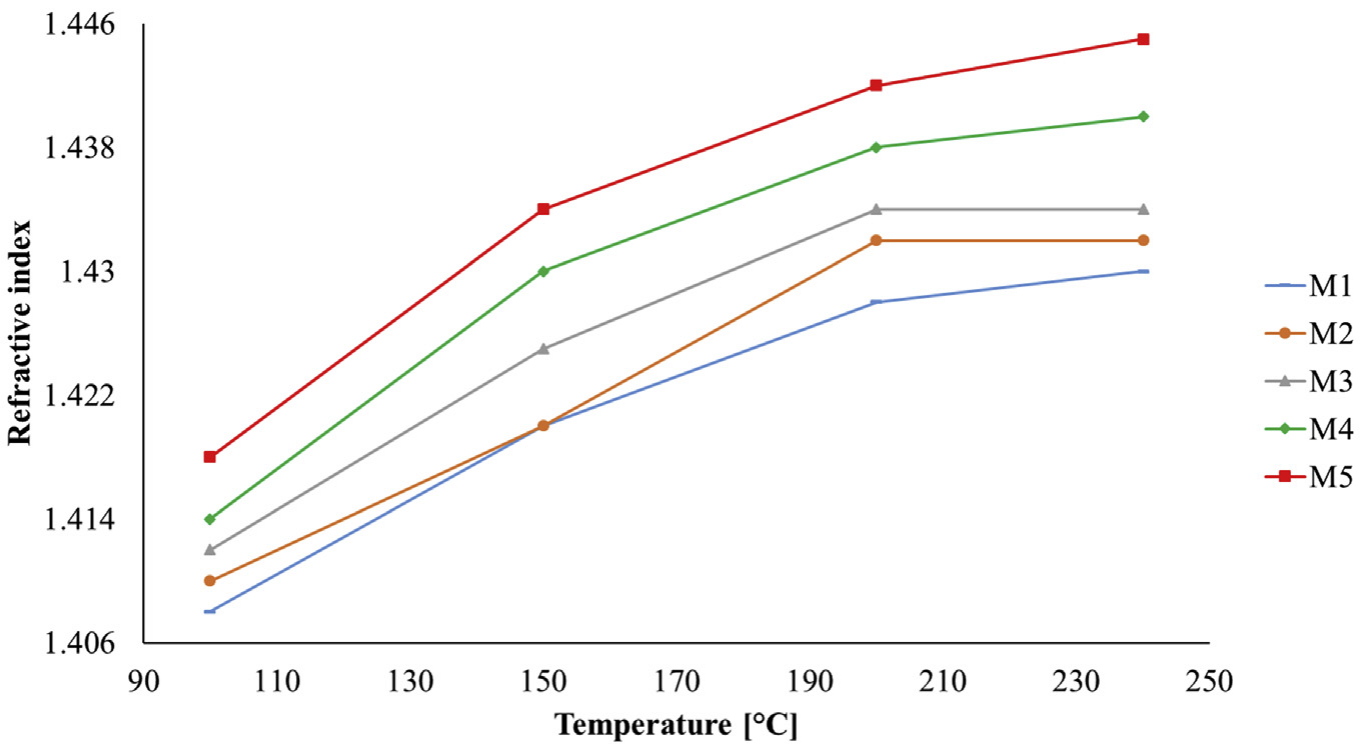
Graphs of the refractive index of the PDMS samples.

The measurements made with the Abbe refractometer determined the refractive indices for each PDMS sample cured at different temperatures and are shown in Figure 6; we observe that an increase in the refractive index of the samples is related to an increase in the variation of the synthesis parameters, as curing temperature and mixture ratio.

**Figure 7:**
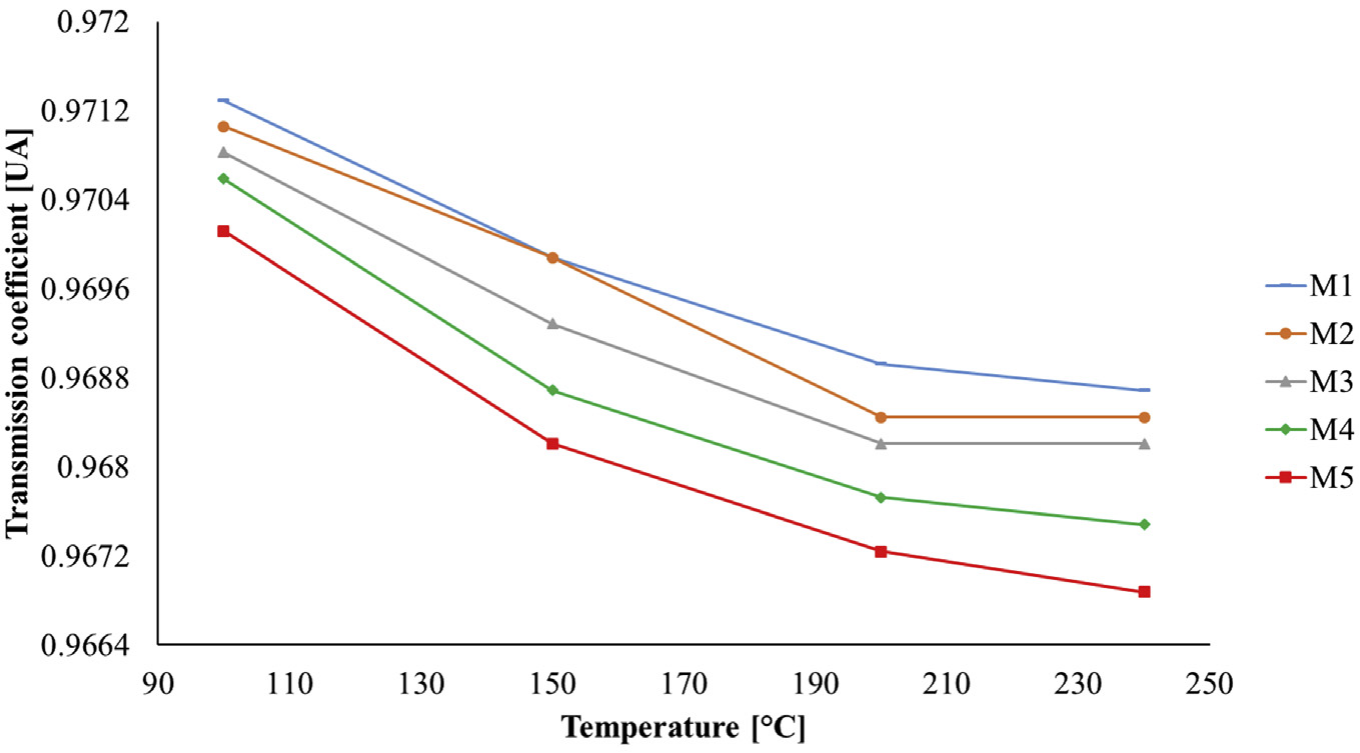
Transmission coefficients of the PDMS samples.

Figure 7 shows the transmission coefficients that were directly calculated from the refraction indices shown in Figure 6; Fresnel formulation with normal incidence was considered in an air-PDMS interface to obtain these results [50].

**Figure 8:**
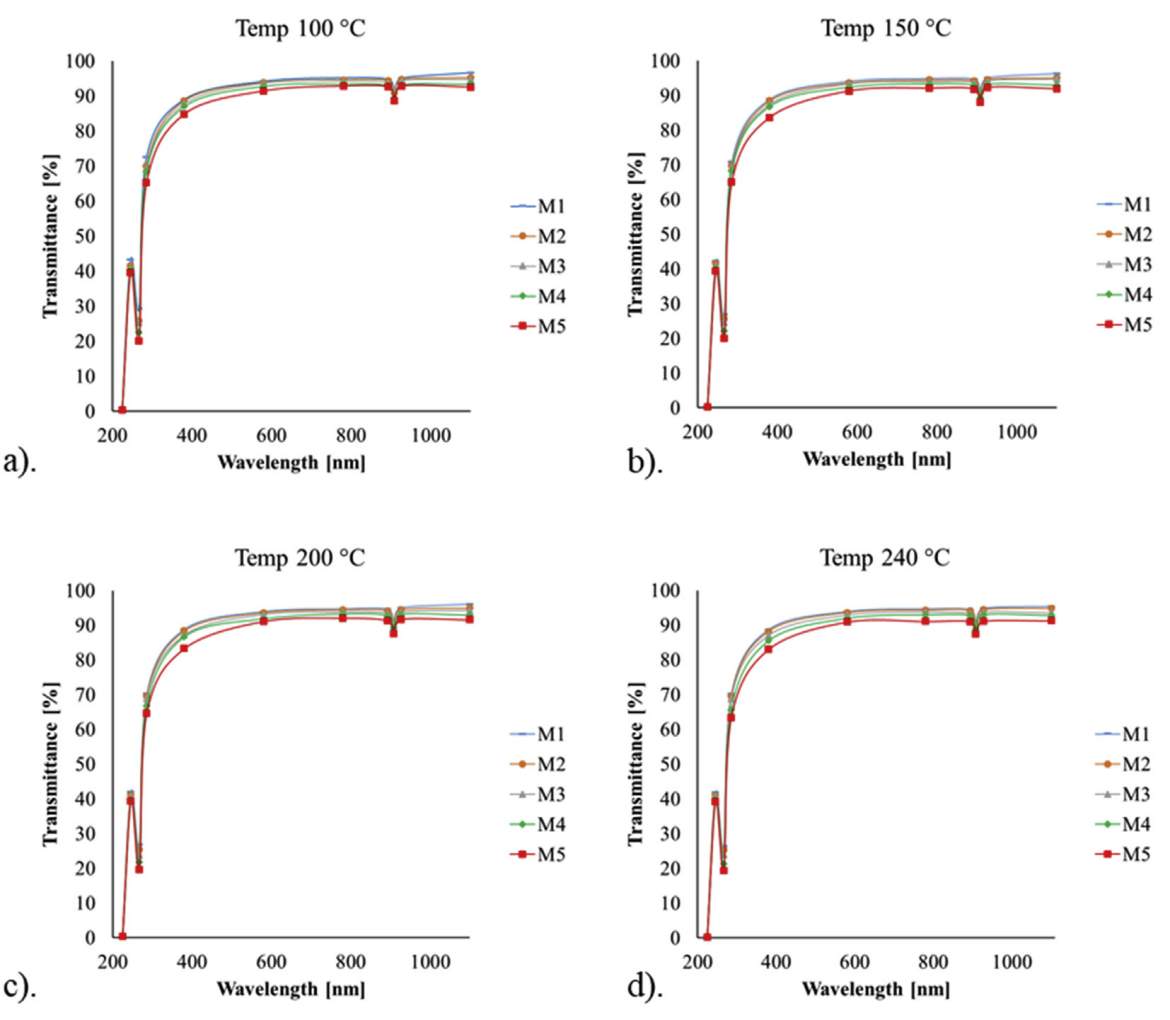
Transmittance spectra of the PDMS samples cured at: a) 100 °C, b) 150 °C, c) 200 °C and d) 240 °C.

The transmittance spectra of the samples at different curing temperatures are shown in Figure 8. An increase of both, catalyst and curing temperature produces a decrease of transmittance in the visible region of the spectrum. Moreover, two absorption peaks are evident for all the samples, one in the UV region of the spectrum at 266 nm and the other in the IR region at 908 nm as seen in the figures.

**Figure 9:**
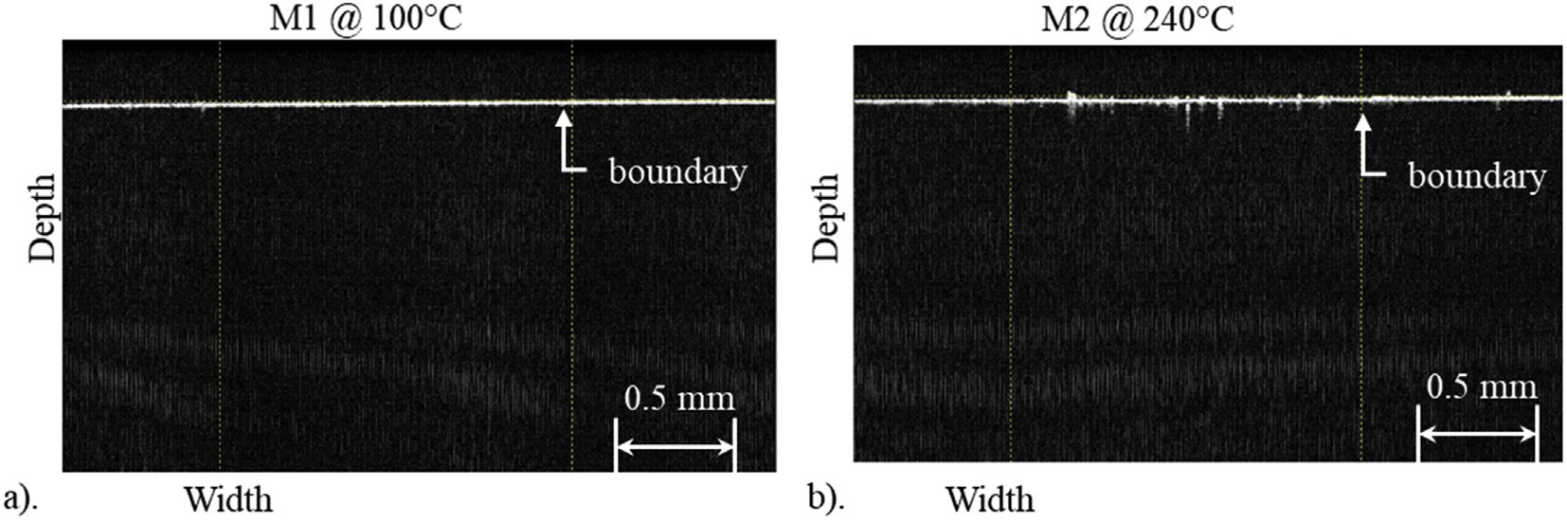
OCT cross-sectional images of the samples: a) M1 at 100 °C and b) M5 at 240 °C.

OCT measurements of the PDMS samples show that no porosity is present, and the samples are homogeneous in a qualitative way as seen in Figure 9, where two cross-sectional images of the scanned samples with different synthesis parameters are shown.

**Figure 10:**
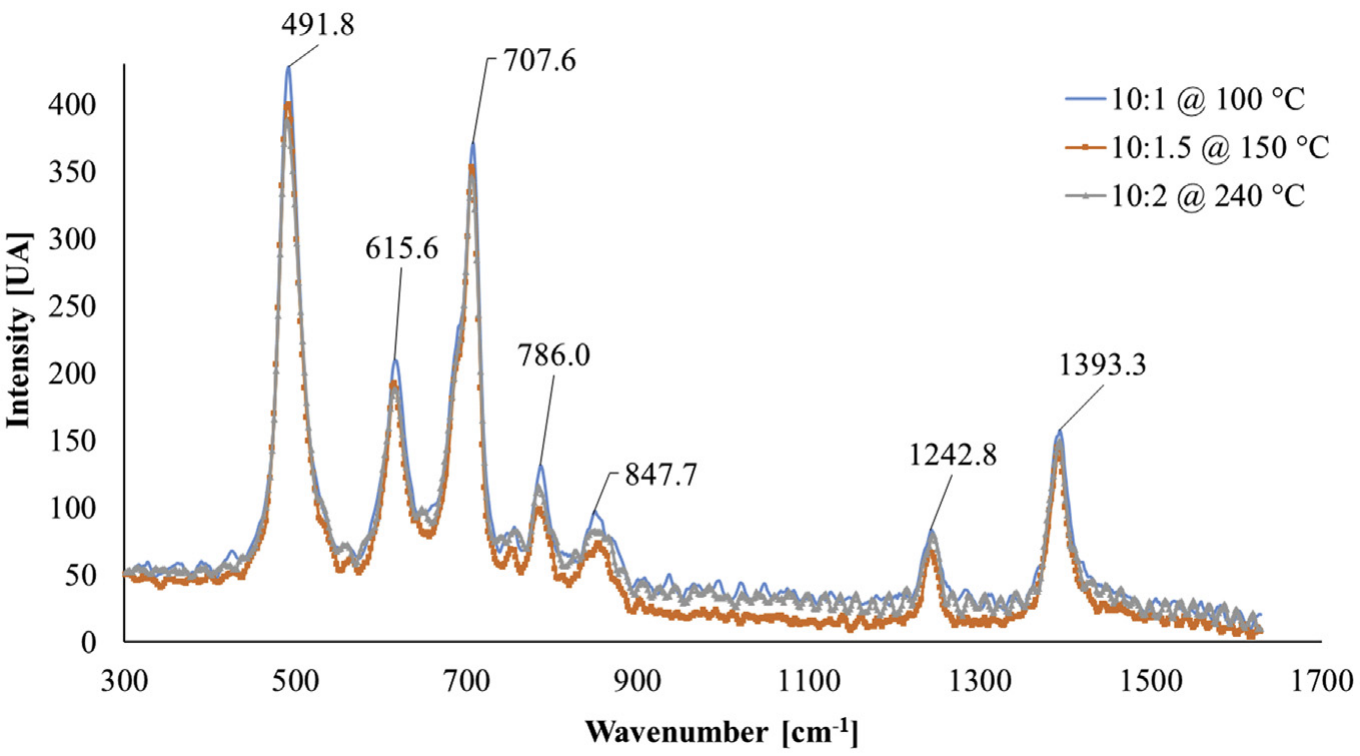
Raman spectra of three different PDMS samples.

Raman spectroscopy studies were performed to the PDMS samples; all the resulted spectra exhibit similar behavior, although visible changes in intensity and width of the peaks are observed for each sample; Figure 10 shows the Raman spectra for three different samples, the range of the selected spectrum is found in half of the evaluated spectra (all samples exhibit approximately the same peaks).

### 3.3 Application: PDMS multi-layered biconic lens

An important application of compiling relevant mechanical and optical properties of the PDMS is to obtain certain optical components with specific features such as multi-layered lenses with different refractive indices.

**Figure 11:**
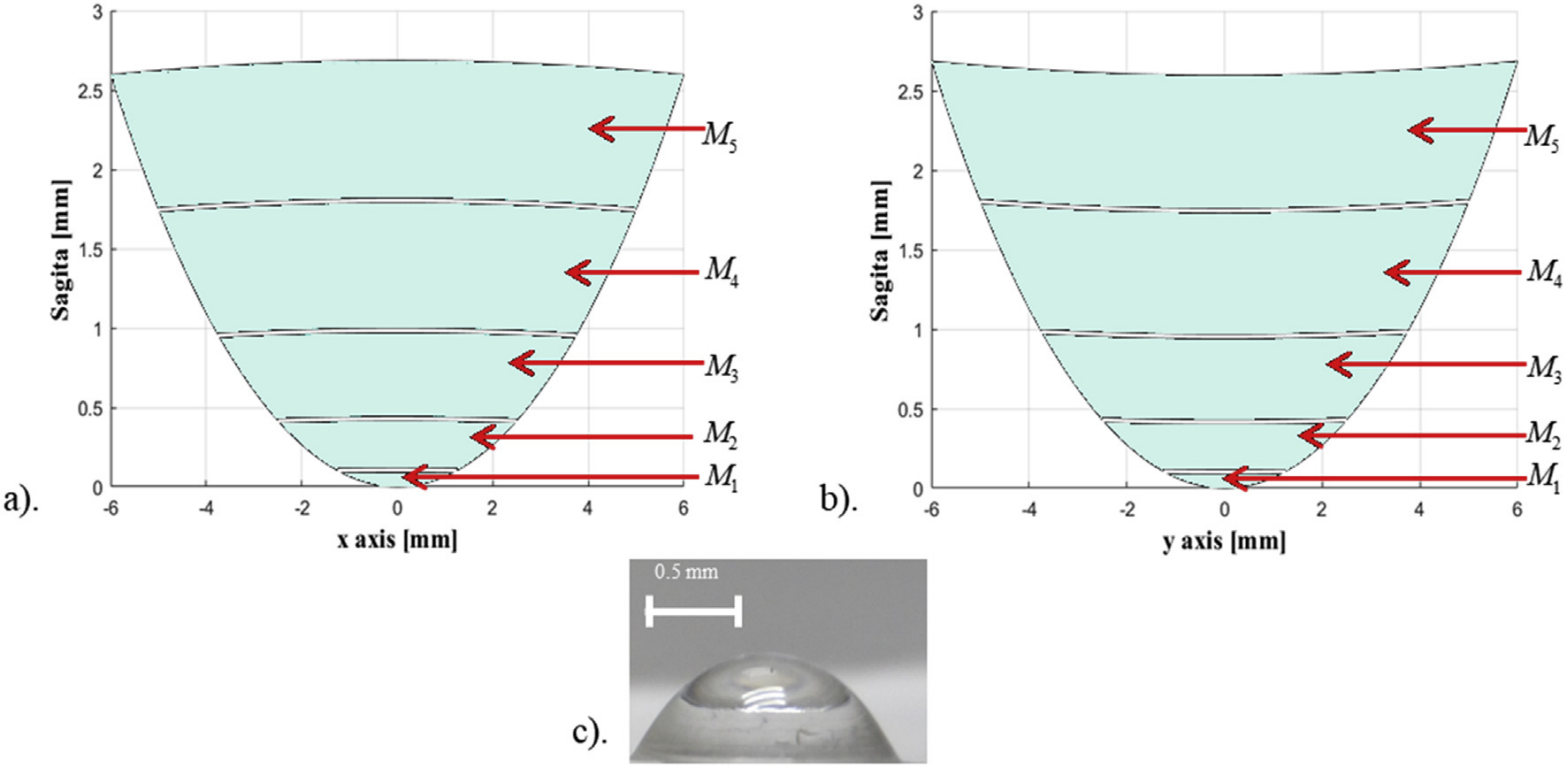
Profile of the biconic refractive component and sectional division of the PDMS layers. Each layer was elaborated using the synthesis parameters shown in Table 1, it was designed with different refractive index and the curing temperature implemented for each layer was 150 °C for 18 min a) x-axis, b) y-axis and c) elaborated tunable lens with multiple layers of PDMS.

Once the mechanical and optical characterizations of the PDMS samples were concluded, a tunable solid elastic lens with biconic profile was modeled with the well-known equation used in optical sciences given by [51]:

(2) $$z\left(x,y\right)=\frac{\frac{x^{2}}{R_{x}}+\frac{y^{2}}{R_{y}}}{1+\sqrt{1-\frac{\left(1+Q_{x}\right)x^{2}}{R_{x}^{2}}+\frac{\left(1+Q_{y}\right)y^{2}}{R_{y}^{2}}}},$$

where *Q*_*x*_ = 1 + *k*_*x*_ and *Q*_*y*_ = 1 + *k*_*y*_; *k*_*x*_*=*-0.465 and *k*_*y*_*=*-0.481 are the conic constants and *R*_*x*_ = 7.63 and *R*_*y*_ = 7.40 are the curvature radii in the *x* and *y* directions respectively. Eq. (2) describes a centered biconic surface where the apex corresponds to the origin of the coordinate system. The resultant profile, shown in Figure 11, was then divided by volumetric sections, where each section corresponds to a PDMS layer elaborated with different synthesis parameters according to the classification from Table 1, in particular we are interested in the elaboration of tunable optics components with certain GRIN distributions, i.e., bio-inspired optical components with application in visual sciences.

## 4 Discussion and conclusions

The mechanical tests performed on manufactured samples with different concentration ratios of the PDMS mixture components and curing parameters, showed that the tension, compression and shear moduli along with its elastic limit, increase as the curing temperature and the concentration of the curing agent (catalyst) in the mixture increase. The obtained experimental values of the mechanical moduli are within ranges and in concordance with those previously published in the literature. In addition, a curve fit was made to the experimental measurements of the elastic modulus and a function that describes their behavior in terms of the curing temperature and mixture ratio was obtained; to authors' knowledge, this is the first time a mathematical expression for this parameter is reported within the described ranges [19, 21, 25].

Regarding to the optical characterization of the samples, the measured refractive indices are within the range of 1.408–1.445, which are comparable to those reported in the literature [22, 27, 29, 36, 40]; from Figure 6, we can see that an increase in the catalyst and/or curing temperature produces an increase of the refractive index of each sample.

Moreover, the results obtained from the transmittance spectra of the PDMS samples indicate a decrease of transmittance with the increase of the mixture ratio and curing temperature, which agrees with the results previously reported [28]. Also, the images from OCT study (Figure 9) show homogeneity and no presence of air bubbles in the samples.

Table 2 shows the maxima found from the Raman spectra and the corresponding vibrational mode for each maximum is mentioned. This confirms that contamination or alteration of material in the synthesis process is not present; the same maxima prevail in all the studied samples. Again, this leads us to the conclusion that the material does not present chemical change in its chains, and the opto-mechanical changes are produced due to polymer hardening. These are caused by the increase in the proportion/ratio of the synthesis parameters and the increase in the curing temperature.

**Table 2:** Vibrational modes of PDMS samples obtained from the Raman spectra [25, 52, 53].

Wavenumber found (cm^−1^)	Vibrational mode
491.8	Stretch Si–O – Si
615.6	Stretch asymmetric Si–C
707.6	Stretch symmetrical Si–C
786.0	Stretch Si–C
847.7	Stretch asymmetric CH_3_
1242.8	Bending symmetrical SiCH_3_
1393.3	Bending asymmetric CH_3_

The data obtained from this characterization has been used to manufacture a low cost tunable multi-layered biconic lens made of PDMS, which has potential applications in robotic systems and visual sciences.

## Declarations

### Author contribution statement

Angel S. Cruz-Félix: Conceived and designed the experiments; Performed the experiments; Analyzed and interpreted the data; Contributed reagents, materials, analysis tools or data; Wrote the paper.

Agustin Santiago-Alvarado: Conceived and designed the experiments; Analyzed and interpreted the data; Contributed reagents, materials, analysis tools or data; Wrote the paper.

Josimar Márquez-García: Performed the experiments; Analyzed and interpreted the data; Contributed reagents, materials, analysis tools or data.

Jorge González-García: Conceived and designed the experiments; Performed the experiments.

### Funding statement

This research did not receive any specific grant from funding agencies in the public, commercial, or not-for-profit sectors.

### Competing interest statement

The authors declare no conflict of interest.

### Additional information

No additional information is available for this paper.

## Data availability

The raw/processed data required to reproduce these findings cannot be shared at this time due to technical or time limitations.
